# Case Report: Atypical hepatobiliary manifestations associated with erythrocyte membrane instability in glucose transporter type 1 deficiency syndrome

**DOI:** 10.3389/fped.2026.1790301

**Published:** 2026-05-20

**Authors:** Hahnbie Lee, Sook Won Ryu, Jeana Hong

**Affiliations:** 1 Kangwon National University School of Medicine, Chuncheon, Republic of Korea; 2 Department of Laboratory Medicine, Kangwon National University Hospital, Chuncheon, Republic of Korea; 3 Department of Pediatrics, Kangwon National Children's Hospital, Chuncheon, Republic of Korea

**Keywords:** cryohydrocytosis, gallstones, glucose transporter type 1, GLUT1 deficiency syndrome, hemolysis

## Abstract

**Background:**

Glucose transporter type 1 (GLUT1) deficiency syndrome (GLUT1DS) is a rare neurometabolic disorder caused by pathogenic variants in *SLC2A1*. While neurological symptoms such as early-onset epilepsy, developmental delay, and movement disorders are well documented, hematologic and hepatobiliary complications are rarely reported, and their underlying mechanisms remain poorly defined.

**Case presentation:**

We report a boy diagnosed with GLUT1DS at five years of age who presented with intractable seizures and persistent indirect hyperbilirubinemia. Genetic testing identified a heterozygous in-frame deletion in *SLC2A1* [c.1306_1308del (p.Ile436del)], confirming the diagnosis of GLUT1DS. A co-occurring heterozygous *UGT1A1* variants including coding and promoter lesions were also identified, initially suggestive of impaired bilirubin conjugation. During follow-up, the patient developed recurrent episodes of jaundice, anemia, and abdominal pain, ultimately attributed to chronic hemolysis with gallstone-related cholestasis requiring endoscopic retrograde cholangiopancreatography. To assess erythrocyte membrane integrity, specialized red blood cell studies were performed at 11 years of age. A temperature-dependent potassium leak test demonstrated cold-enhanced cation leakage consistent with pseudohyperkalemia and cryohydrocytosis, providing functional evidence of erythrocyte membrane instability associated with GLUT1 dysfunction.

**Conclusion:**

This case expands the phenotypic spectrum of GLUT1DS by linking erythrocyte membrane instability to recurrent hemolysis and rarely reported hepatobiliary complications. Functional evaluation of erythrocyte membrane integrity may facilitate recognition of atypical systemic manifestations of GLUT1DS and supports the need for multidisciplinary surveillance beyond neurological care.

## Introduction

Glucose transporter type 1 (GLUT1) deficiency syndrome (GLUT1DS) is a rare genetic disorder caused by pathogenic variants in *SLC2A1*, which encodes GLUT1, a protein essential for glucose transport across the blood–brain barrier. GLUT1 plays a critical role in brain energy metabolism, and its deficiency leads to a state of cerebral energy deprivation. Since its first description in 1991, the clinical features of GLUT1DS have been well characterized, primarily presenting as neurological manifestations, including early-onset seizures, movement disorders, intellectual disability, and hypoglycorrhachia (1). A Ketogenic diet (KD) is the standard therapy to improve neurological symptoms by providing an alternative cerebral fuel source (2).

Beyond these hallmark neurological features, atypical and extra-neurological manifestations of GLUT1DS have increasingly been recognized, as highlighted in recent expert recommendations (2). These rare systemic features have been attributed to GLUT1 dysfunction in non-neuronal tissues, including erythrocyte membranes, however, the long-term course and optimal management of these atypical manifestations remain insufficiently defined (3).

Here, we report a Korean boy diagnosed with GLUT1DS beyond toddlerhood presented with rarely reported hepatobiliary complications along with evidence of erythrocyte membrane instability. This case expands the phenotypic spectrum of GLUT1DS, providing clinically relevant insights into the need for hepatobiliary surveillance and multidisciplinary care beyond the typical neurological presentation.

## Case presentation

A 5.5-year-old boy was referred to pediatric gastroenterology for evaluation of elevated serum bilirubin levels identified during admission for intractable seizure. He was born at 37 + 5 weeks of gestation with a birth weight of 2.5 kg and underwent exchange transfusion for severe neonatal jaundice on day 3 of life. He was subsequently diagnosed with congenital cataracts and developmental delay. Brain magnetic resonance imaging performed during the neonatal period demonstrated periventricular leukomalacia. Since 10 months of age, he had experienced multiple hospitalizations for recurrent seizures that remained poorly controlled despite treatment with multiple antiepileptic medications.

**Figure 1:**
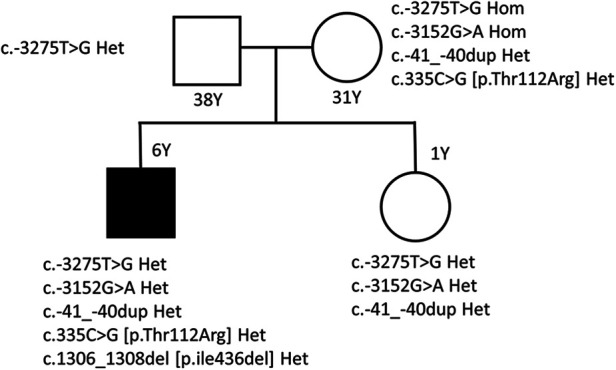
Hepatobiliary findings on magnetic resonance cholangiopancreatography. Magnetic resonance cholangiopancreatography performed at nine years of age demonstrates gallstones within the gallbladder and common bile duct, with associated dilatation of the extrahepatic bile duct. Concomitant splenomegaly is also observed, consistent with chronic hemolysis.

At the time of referral, he weighed 17 kg (8th percentile for age) and was wheelchair-dependent, with severe spasticity, global developmental delay, and impaired verbal communication, requiring full assistance with feeding. Laboratory tests showed total and direct bilirubin (TB and DB) levels of 2.5 (normal range, 0.2–1.2) and 1.1 (normal range, 0–0.4) mg/dL, respectively, with normal hemoglobin (Hb), transaminases, and electrolyte levels (Table 1). Abdominal ultrasonography demonstrated mild splenomegaly (10 cm) without hepatobiliary abnormalities. During the evaluation of his hyperbilirubinemia and intractable seizures, whole-exome sequencing identified a heterozygous NM_006516.3:c.1306_1308del (p.Ile436del) variant in *SLC2A1*, classified as likely pathogenic according to ACMG criteria (PS3, PM4, PM1, PM2), confirming the diagnosis of GLUT1DS. In addition, four heterozygous variants in *UGT1A1* were detected including a coding variant of NM_000463.3:c.335C > G (p.Thr112Arg), which is classified as a variant of uncertain significance and 3 promoter variants (NM_000463.3:c.-3275T > G, NM_000463.3:c.-3152G > A, and NM_000463.3:c.-41_40dup), all suggestive of impaired bilirubin conjugation commonly observed in Gilbert syndrome. Whole-exome sequencing was initially performed in the proband and targeted Sanger sequencing for the identified *SLC2A1* and *UGT1A1* variants were subsequently performed in both parents and his sibling. The *SLC2A1* variant was not detected in any of the tested family members (Figure 1), suggesting that the variant likely occurred *de novo*, although germline mosaicism cannot be entirely excluded. A KD was initiated immediately after diagnosis of GLUT1DS. Over the subsequent two years, TB levels ranged from 1.7 to 3.5 mg/dL, with stable Hb levels and persistently normal transaminases.

**Table 1:** Longitudinal clinical and laboratory findings in relation to major clinical events.

Time point	Age (years)	Hb (g/dL)	MCV (fL)	Reticulocyte count (%)	TB (mg/dL)	DB (mg/dL)	Clinical events/Interventions
Initial presentation	5.5	13.4	101.8		2.5	1.1	Abdominal ultrasonography showing splenomegaly; Genetic analysis performed
First admission	9.2	11.3	104.9	12.2	14.6	9.3	MRCP showing GB and CBD stones; UDCA initiated
Second admission	11.2	13.2	93.6	5.1	27.4	19.2	ERCP with stone removal
Third admission	11.5	9.3	103.7	11.9	6.4	1.4	Cation leakage test performed
Follow-up	11.6	12.1	92.7	6.5	2.7	0.8	

Hb, hemoglobin; MCV, mean corpuscular volume; TB, total bilirubin; DB, direct bilirubin; MRCP, magnetic resonance cholangiopancreatography; GB, gallbladder; CBD, common bile duct; UDCA, ursodeoxycholic acid; ERCP, endoscopic retrograde cholangiopancreatography.

**Figure 2:**
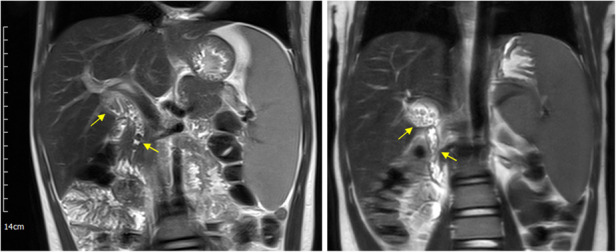
Longitudinal changes in laboratory parameters during the clinical course. Serial laboratory findings plotted by age illustrate episodes of cholestatic hepatitis and hemolytic crises, as reflected by changes in total bilirubin (TB, dark blue bars), direct bilirubin (DB, light blue bars), hemoglobin levels (Hb, red line with circles; g/dL), and reticulocyte counts (black crosses; %).

At nine years of age, he presented again with several months of nausea and new-onset jaundice. The KD had been discontinued two years earlier because of adherence difficulties. Moreover, physical therapy had been withheld because the mother perceived an association with increased seizure activity. Laboratory tests revealed mild anemia [Hb 11.3 (normal range, 12–14) g/dL], macrocytosis [mean corpuscular volume 104.9 (normal range, 77.8–91.1) fL], reticulocytosis, and cholestatic hepatitis (Table 1). Peripheral blood smear (PBS) demonstrated stomatocytes, and echinocytes, accompanied by markedly reduced haptoglobin levels and elevated plasma Hb, consistent with hemolysis. Magnetic resonance cholangiopancreatography showed gallbladder and common bile duct (CBD) stones with ductal dilatation (Figure 2). His symptoms and cholestasis improved with following treatment with ursodeoxycholic acid, and folate supplementation was initiated for presumed chronic hemolysis.

**Figure 3:**
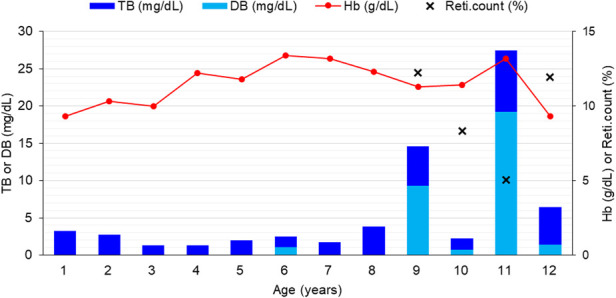
Genetic analysis and familial segregation of identified *SLC2A1* and *UGT1A1* variants. Sanger sequencing chromatograms demonstrating the heterozygous *SLC2A1* NM_006516.3:c.1306_1308del (p.Ile436del) variant in the proband, which was not detected in either parent or the sibling, supporting a likely *de novo* occurrence.

At 11 years of age, he was re-admitted with abdominal pain and severe cholestatic hepatitis, with TB and DB levels of 27.4 and 19.2 mg/dL, respectively, and aspartate and alanine aminotransferase (AST and ALT) levels of 51 (normal range, 13–33) and 119 (normal range, 8–42) U/L, respectively (Table 1, Figure 3). With a further increase in bilirubin (TB/DB 30.8/22.2 mg/dL), endoscopic retrograde cholangiopancreatography was performed with CBD stone removal and stent placement, which resulted in clinical improvement and normalization of liver enzyme levels. Six months later, he returned with icteric sclera and an anemic appearance following a febrile respiratory infection. Laboratory tests revealed anemia (Hb 9.3 g/dL) and TB/DB was 6.4/1.4 mg/dL, while AST and ALT remained within normal ranges, compatible with another episode of hemolysis (Table 1, Figure 3).

**Figure 4:**
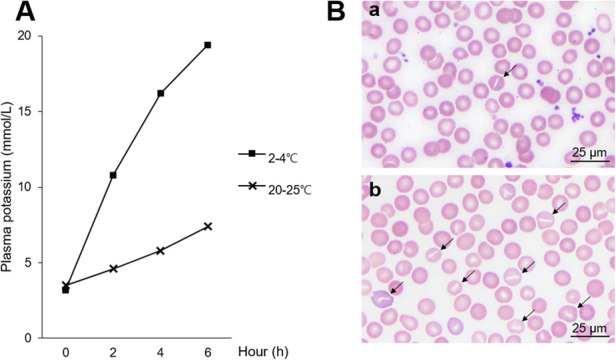
Temperature-dependent potassium leak test and erythrocyte morphology. **(A)** Time-dependent changes in plasma potassium concentration in heparinized whole blood stored at room temperature (20–25 °C) and under refrigerated conditions (2–4 °C). Plasma potassium levels increase over time, with a more pronounced rise during refrigerated storage, consistent with temperature-dependent pseudohyperkalemia. **(B)** Peripheral blood smear images obtained six hours after blood collection: **(a)** Room temperature storage showing relatively preserved erythrocyte morphology; **(b)** Refrigerated storage showing increased stomatocytosis (black arrows), consistent with erythrocyte membrane instability.

To investigate the cause of his recurrent hemolysis, erythrocyte membrane studies were performed. A temperature-dependent potassium leak test, conducted as previously described, demonstrated progressive elevation in plasma potassium levels over time at both room temperature (20–25 ℃) and refrigerated storage (2–4 ℃), with a more prominent increase under refrigerated conditions (4, 5) (Figure 4). These findings confirmed pseudohyperkalemia and cryohydrocytosis resulting from temperature-sensitive cation leakage associated with underlying erythrocyte membrane instability. PBS after cold storage also showed an increased proportion of stomatocytes compared with room temperature storage (Figure 4).

## Discussion

This case highlights an under-recognized systemic manifestation of GLUT1DS, characterized by recurrent hemolysis and clinically significant hepatobiliary complications associated with erythrocyte membrane instability. Although GLUT1DS is primarily defined by neurological features, this patient expands the phenotypic spectrum by linking a pathogenic *SLC2A1* variant to functional erythrocyte membrane dysfunction with subsequent hepatobiliary morbidity. This systemic presentation emphasizes the critical role of GLUT1 in non-cerebral tissues and highlights the potential for long-term morbidity related to extra-neurological complications.

In contrast to the well-characterized neurological features of GLUT1DS, hepatobiliary morbidity attributable to chronic hemolysis has been rarely reported in the literature. This case provides clinically relevant evidence that sustained hemolysis in GLUT1DS can lead to progressive hepatobiliary complications. Notably, to our knowledge, this represents the first reported case of gallstone formation requiring endoscopic intervention in a patient with GLUT1DS. Moreover, our patient posed a significant diagnostic challenge due to the coexistence of a pathogenic *SLC2A1* c.1306_1308del (p.Ile436del) variant and several *UGT1A1* variants. The *UGT1A1* variants could theoretically account for chronic indirect hyperbilirubinemia consistent with Gilbert syndrome and potentially contribute to gallstone formation (6). In particular, the presence of the promoter variants as c.-3275T > G, and c.-41_40dup which are known regulatory polymorphisms with reduced *UGT1A1* transcriptional activity and commonly observed in Korean Gilbert syndrome, led us to initially consider impaired bilirubin conjugation as a contributing factor of his persistent indirect hyperbilirubinemia and gallstone formation. However, the subsequent emergence of marked stomatocytosis on PBS, reticulocytosis, and low haptoglobin levels was not fully explained by the *UGT1A1* variants, prompting further evaluation of recurrent hemolysis episodes as the primary driver of hepatobiliary morbidity. Accordingly, erythrocyte membrane functional testing was performed. Based on these findings, the *UGT1A1* variants are considered modifiers of bilirubin metabolism rather than causative factors, whereas hemolysis represents the predominant pathogenic mechanism. The underlying mechanism of the hematologic features rarely described in GLUT1DS is plausibly explained by mutation-associated erythrocyte membrane instability. Prior evidence suggests that hemolysis in GLUT1DS is caused by a dual functional defect: impaired glucose transport and pathological cation leakage due to tertiary structural alterations of GLUT1 with widening of pore region in the erythrocyte membrane (7). This combined functional and structural compromise, rather than erythrocyte glucose deprivation alone, drives erythrocyte fragility and underlies cryohydrocytosis, which is characterized by leakage of sodium and potassium ions from erythrocytes at low temperatures (0–4 ℃). Although GLUT1DS is not classified as a hereditary stomatocytosis disorder, a group of red cell membrane disorders, both conditions share features of altered erythrocyte cation permeability leading to membrane instability and hemolysis. Recent advances have highlighted the role of membrane transport abnormalities and channelopathies in these conditions, providing mechanistic insight into temperature-dependent cation leakage, erythrocyte morphological changes and its clinical manifestations (8, 9).

Genotype–phenotype correlations may further explain the rarity of this presentation. In a recent Asian study involving 30 Chinese children with GLUT1DS, none exhibited hematologic or hepatobiliary complications (10). Moreover, a few reports about the clinical characteristics of Korean children with GLUT1DS described only neurological manifestations (11, 12). The *SLC2A1* c.1306_1308del (p.Ile436del) variant identified in our patient has not been reported in these cohorts and, to our knowledge, represents the first Korean case associated with this variant. In contrast, several published cases describing anemia, hemolysis, pseudohyperkalemia, or cryohydrocytosis have frequently involved a subset of *SLC2A1* variants affecting the C-terminal transmembrane domains of GLUT1, particularly in-frame deletions (5, 13). Notably, patients harboring these variants share overlapping features with our case, including neonatal jaundice, chronic hemolysis, erythrocyte morphological abnormalities, and temperature-dependent cation leakage (3, 14). Accordingly, we summarize previously reported cases with *SLC2A1* variants associated with cryohydrocytosis-like phenotypes, including our patient, in Table 2 (3–5, 13–15). Collectively, these observations suggest that patients harboring specific transmembrane *SLC2A1* variants predispose to erythrocyte membrane instability and present with this distinctive hematologic phenotype.

**Table 2:** Summary of reported GLUT1 deficiency syndrome with cryohydrocytosis-like phenotypes.

Author (year)	Age/Sex	*SLC2A1* variant	Neurological phenotype	Hematologic feature				Hepato-biliary involvement	Systemic features	Pseudo hyper-kalemia	Cation leakage test
				Anemia	Reticulo-cytosis	Macro-cytosis	Hypo-haptoglobinemia				
Fricke et al^a^ (2004) (15)	Adult/M	p.Gly286Asp	Seizures, Intellectual disability, Cerebellar ataxia, Hydrocephalus	**−**	**−**	**+**	**+**	Hepatomegaly, Increased bilirubin and transaminase	Cataracts, Dysmorphic feature, Splenomegaly	**+**	Performed
Flatt et al^b^(2011) (5)	Adult/F	p.Ile435 or 436del	Seizures, Intellectual disability, Spastic quadriplegia	**+**	**+**	**+**	NR	Hepatomegaly	Cataracts, Splenomegaly	**+**	Performed
Weber et al. (2008) (13)	60Y/F	p.Q282_S285del	Paroxysmal exertion-induced dyskinesia	**−**	NR	**+**	**+**	Increased bilirubin and transaminase	Splenomegaly	**+**	Performed
	40Y/M	p.Q282_S285del	Paroxysmal exertion-induced dyskinesia	**−**	**+**	**+**	**+**	Increased bilirubin and transaminase	Splenomegaly	**+**	Performed
	15Y/M	p.Q282_S285del	Paroxysmal exertion-induced dyskinesia, Seizures, Developmental delay	**+**	**+**	**+**	**+**	Increased bilirubin	Splenomegaly	**+**	Performed
	9Y/M	p.Q282_S285del	Seizures, Developmental delay	**+**	**+**	**+**	**+**	Increased bilirubin	Splenomegaly	**+**	Performed
Bawazir et al. (2012) (4)	Infant/F	p.Ile435 or 436del	Seizures, Intellectual disability, Developmental delay, Periventricular calcification	**±**	**+**	**+**	**+**	Increased bilirubin and transaminase	Neonatal jaundice, Cataracts, Retinal dysfunction, Splenomegaly	**+**	Performed
Shibata et al. (2017) (14)	1.8Y/F	p.Ile436del	Seizures Periventricular calcification and brain atrophy on MRI	**−**	**+**	**+**	**+**		Cataracts	9.3^c^ to 4.7^d^ mmol/L	Not done
Furia et al. (2023) (3)	15Y/M	p.Ile436del	Seizure, Intellectual disability	NR	NR	NR	NR		Neonatal jaundice, Cataracts, Splenomegaly	5.8∼7.2 mmol/L	Not done
Current study	5.5Y/M	p.Ile436del	Seizures, Developmental delay	**+**	**+**	**+**	**+**	Cholelithiasis, Increased bilirubin and transaminase	Neonatal jaundice, Cataracts, Splenomegaly	**+**	Performed

a Original clinical report.

b Genetic confirmation paper.

c Serum potassium level 3 h after blood sampling.

d Serum potassium level immediately after blood sampling.

NR, not reported; ± Hb was normal or lower normal.

From a clinical perspective, this case highlights several practical considerations in the management of patients with GLUT1DS. In our patient, hemolytic episodes appeared to be precipitated by physiological stressors, including intercurrent infection, cold exposure, and strenuous physical therapy, consistent with the temperature-sensitive nature of the demonstrated cation leak. We recommended the parents avoidance of intercurrent infections when possible, minimizing cold exposure, and limiting excessive physical stress. Since then, the patient has remained clinically stable for more than one year under outpatient follow-up, with total bilirubin levels ranging from 1.8 to 4.2 mg/dL, normal direct bilirubin and transaminase levels, and stable hemoglobin levels without further hospitalizations. From a procedural perspective, careful peri-procedural management may also be important in patients with GLUT1DS. In our case, prolonged fasting was minimized and metabolic status was closely monitored during endoscopic intervention, which may have contributed to the absence of procedure-related complications.

In addition, recognition of pseudohyperkalemia is critical in this context. Careful attention to blood sample handling, including avoidance of prolonged storage and prompt processing, may prevent misinterpretation of potassium measurements and avert unnecessary interventions (3, 4, 14). In patients with suspected erythrocyte membrane instability, pre-analytical factors are critical for accurate interpretation of potassium levels. Delayed processing or storage at low temperatures may lead to pseudohyperkalemia due to temperature-dependent cation leakage. Therefore, careful attention to sample handling and timely processing is essential to avoid misinterpretation.

Finally, although KD therapy is primarily indicated for the control of neurological symptoms, its potential impact on extra-neurological manifestations remains uncertain (2). It is conceivable that sustained metabolic stability under KD might influence systemic manifestations; however, this remains speculative and cannot be inferred from a single case. Given the temporal association between prolonged discontinuation of KD and the subsequent development of hepatobiliary complications in our patient, the therapeutic role of sustained KD therapy in modulating erythrocyte instability warrants further investigation.

In conclusion, this report broadens the clinical spectrum of GLUT1DS by demonstrating that erythrocyte membrane instability can lead to recurrent hemolysis with downstream hepatobiliary complications. From a clinical standpoint, multidisciplinary surveillance—including hemolysis evaluation, hepatobiliary monitoring, and careful attention to triggering factors and specimen handling—may be essential to mitigate long-term morbidity in this subgroup of GLUT1DS patients, particularly those harboring *SLC2A1* variants associated with erythrocyte membrane instability.

## Data Availability

The raw data supporting the conclusions of this article will be made available by the authors, without undue reservation.
